# Internet-Based Problem Management Plus Intervention for Antenatal Depression: Randomized Controlled Trial

**DOI:** 10.2196/81998

**Published:** 2026-03-27

**Authors:** Shuanghui Zheng, Shiyu Wang, Xiaoyu Shui, Jing Zhao, Ying Lin, Qian Huang, Siqi Li, Yang Chen, Zhijie Zou, Jin Zheng, Xiaoli Chen

**Affiliations:** 1 School of Nursing, Wuhan University, Building 2, School of Medicine, Wuhan University, 4th Fl, No. 115, Donghu Road, Wuhan, 430071, China, 86 13437157662; 2 Department of Nursing, Maternal and Child Health Hospital of Hubei Province, Wuhan, China; 3 Qianjin 4th Road Campus, Hospital of Stomatology, Wuhan University, Wuhan, China

**Keywords:** antenatal depression, problem management plus, PM+, randomized controlled trial, digital intervention, mental health

## Abstract

**Background:**

The identification and management of depression during pregnancy is an important public health issue. Although many existing psychological intervention programs are effective, their implementation is plagued by issues, such as insufficient professional resources and lengthy intervention cycles. Studies have suggested that internet-based problem management plus (IPM+) can effectively address the aforementioned challenges in the management of general depression. However, its application in the pregnant population remains to be verified.

**Objective:**

The objective of our study was to explore the effect of IPM+ on depressive symptoms in pregnant women.

**Methods:**

From April to October 2024, 80 pregnant women were recruited from the Hubei General Hospital and randomly assigned to the intervention group (n=40) and the control group (n=40). The control group received routine care, while the intervention group received a 5-week IPM+ intervention (1.5 hours per week) in addition to routine care. The primary outcome measure is depressive mood, and the secondary outcome measures include anxiety symptoms, perceived stress, and sleep quality. The data were collected using the Edinburgh Postnatal Depression Scale, Pregnancy Anxiety Questionnaire, 4-item Perceived Stress Scale, and Insomnia Severity Index at baseline (T1), immediately after the intervention (T2), and at 3 months (T3).

**Results:**

Compared with the control group, the intervention group showed statistically greater improvements in depressive symptoms (between-group effect size: Hedges *g*=0.74, 95% CI 0.23-1.24 at T2; Hedges *g*=0.76, 95% CI 0.25-1.28 at T3), anxiety symptoms (between-group effect size: Hedges *g*=0.51, 95% CI 0.03-0.98 at T2; Hedges *g*=0.52, 95% CI 0.04-0.99 at T3), and perceived stress (between-group effect size: Hedges *g*=0.71, 95% CI 0.23-1.18 at T2; Hedges *g*=0.62, 95% CI 0.15-1.11 at T3) at both T2 and T3. In terms of sleep quality, the intervention group demonstrated statistically greater improvement compared with the control group only at T3 (between-group effect size: Hedges *g*=0.68, 95% CI 0.19-1.17).

**Conclusions:**

IPM+ effectively alleviates depression and anxiety symptoms, reduces perceived stress, and improves sleep quality in pregnant women with antenatal depression. The intervention effects are sustained for up to 3 months postintervention.

## Introduction

Antenatal depression (AND) refers to depressive episodes occurring during pregnancy (from conception to before delivery) and is considered a clinical subtype of perinatal depression [1]. The global prevalence of AND is reported to be approximately 20.7%, with significant regional variations: the prevalence in low- and middle-income countries (30.3%) is substantially higher than in high-income countries (18.1%) [2]. The prevalence of prenatal depression among women in China (19.7%) has exceeded the average level in high-income countries [3]. If left untreated, it can result in a dual crisis for maternal and infant health. Mothers may experience obstetric complications, such as hyperemesis gravidarum, preeclampsia, miscarriage, preterm birth, and postpartum depression [4]. In severe cases, suicidal ideation or infanticide may occur [5]. For the offspring, long-term negative effects, including cognitive developmental delays and emotional regulation disorders, are possible, and there is a risk of intergenerational transmission of mental health problems [6-9].

Psychotherapy has now become the first-line treatment for AND [10], including evidence-based methods, such as cognitive behavioral therapy (CBT), interpersonal psychotherapy (IPT), and mindfulness-based interventions [11]. Existing evidence suggests that these therapies significantly reduce depressive symptoms and concurrently improve anxiety, sleep quality, and social support levels [12-15]. However, traditional psychotherapy is confronted with 3 core dilemmas: one lies in the barrier of human resources, as its implementation highly relies on highly qualified professionals, involving lengthy training periods and exorbitant labor costs; another manifests as the intervention is excessively time-consuming for participants, which is hardly compatible with the fragmented and mobile schedules of perinatal women, directly resulting in a high dropout rate [16]; and a deeper obstacle stems from sociocultural factors, where the prevalence of stigma leads to only 28.5% of patients with postpartum depression actively seeking professional support [17].

Internet-based problem management plus (IPM+) may become a breakthrough strategy to improve the accessibility of psychological service. Problem management plus (PM+), developed by the World Health Organization (WHO) [18], is a low-intensity intervention that integrates CBT and problem-solving therapy. It not only features transdiagnostic applicability, short duration, and implementability by nonprofessionals (such as community nurses and volunteers) but also consists of 4 modules: stress management, problem-solving–oriented action planning, continuous behavioral activation, and interpersonal relationship enhancement [19]. Several randomized controlled trials have demonstrated that PM+ has significant effects on depression, posttraumatic stress disorder, and anxiety in the general population, with effects lasting up to 3 months postintervention [20-23]. At the same time, compared to traditional offline methods, online psychological interventions offer 3 main advantages: overcoming spatial limitations by relying on various digital platforms, allowing perinatal women to receive interventions anywhere; time flexibility, with asynchronous courses (eg, prerecorded videos, artificial intelligence question and answer) that fit into fragmented schedules; and stigma reduction, as the anonymous environment encourages help-seeking behavior [24]. IPM+ not only boasts the advantages of internet interventions, such as geographical reach, time flexibility, and reduction of stigma, but also retains the strengths of PM+ as an integrated multimodule intervention. However, the application of PM+ in pregnant populations remains in the exploratory stage, since only 1 cultural adaptation study conducted in Pakistan worldwide has involved this [23].

To explore the intervention effect of IPM+ on AND, this study developed a randomized controlled trial, which includes a 5-week intervention period and a 3-month follow-up period. From the perspective of empirical research, it aims to answer the scientific question of “whether IPM+ can improve AND.”

## Methods

### Study Design

This study adopts a single-blind, 1:1 parallel-group randomized controlled trial design. The study was conducted at Hubei General Hospital, a large Grade A tertiary hospital located in Wuhan, China, and strictly followed the CONSORT (Consolidated Standards of Reporting Trials; Checklist 1) guidelines.

### Ethical Considerations

The research was approved by the Ethics Committee of the Medical School of Wuhan University (WHU-LFMD-IRB2024006) and registered with the Chinese Clinical Trial Registry (ChiCTR2400083324). All participants provided written informed consent before completing the baseline assessment. To ensure participant privacy and confidentiality, all collected data were deidentified and stored on a secure, password-protected server, with access strictly restricted to the research team. Regarding compensation, no financial or material incentives were provided to the participants for completing the intervention or the assessment sessions.

### Participants

Participants were recruited through convenience sampling from the Obstetrics Department of Hubei General Hospital between April 2024 and October 2024. Eligible patients who met the inclusion criteria were invited to participate in the study. The inclusion criteria were as follows: (1) age 18 years and above, (2) gestational age of 28 weeks or less, (3) singleton pregnancy, (4) no plans for elective termination of pregnancy, (5) Edinburgh Postnatal Depression Scale (EPDS) score of 10 or higher (if EPDS score of 13 or higher, further assessment was required to determine suitability for enrollment), and (6) ability to understand the study questionnaire. The exclusion criteria were as follows: (1) presence of suicidal thoughts or behaviors, (2) serious organic diseases or functional disorders, (3) severe mental or cognitive disorders (eg, severe intellectual disability, dementia), (4) substance or alcohol dependence or abuse behaviors within the past 6 months, and (5) previous experience with PM+ treatment or simultaneous participation in other psychological interventions.

### Blinding

Non-researchers generated a random number sequence using a random number table, which was then imported into the Excel software and arranged in ascending order. Based on the sorted random number sequence, the first 50% (n=40) of the participants were assigned to the intervention group, and the remaining 50% (n=40) were assigned to the control group. To ensure allocation concealment, the random allocation scheme was sealed in opaque envelopes, numbered sequentially. These envelopes were opened by research assistants, who were not involved in the study evaluation, after the participants completed the baseline data collection to execute the final group assignments. The study adopted a single-blind design, with all participants unaware of their group allocation. Throughout the recruitment, informed consent, and the entire study process, we consistently utilized the unified project title “Perinatal Mental Health Online Support Project.” All participants were informed that the project involved 2 different forms of online guidance programs, both of which were beneficial for pregnancy health. Furthermore, we ensured that both the control group and the intervention group included weekly one-on-one meetings with an online facilitator to maintain consistency. Due to the nature of the intervention, the implementers were not blinded.

### Intervention Group

On the basis of routine care, the intervention group received a 5-week IPM+ intervention (1.5 hours per week). The intervention plan was compiled based on the “PM+” manual of the Chinese version of the WHO [25] and tailored to address the specific characteristics of perinatal depression. Key adaptations included pregnancy-specific case examples and stressors, the incorporation of safe and appropriate activities for the antenatal period, and an emphasis on social support derived from peer interactions among pregnant women. The adapted protocol was reviewed and validated by a multidisciplinary panel of experts (including a clinical psychologist, an obstetrician, a PM+ supervisor, and a senior midwife) for cultural relevance, safety, and feasibility. Furthermore, a usability pretest was conducted with 4 pregnant women (who met the inclusion criteria but did not participate in the main trial) to preliminarily assess its acceptability and comprehensibility. The intervention was conducted in an individualized online format, with structured 1.5-hour sessions every week via the WeChat platform. The intervention was delivered by 2 nonspecialist providers, both of whom were nursing graduate students. Prior to study initiation, they completed a standardized 40-hour training program based on the Chinese version of the WHO PM+ manual [25]. Competency was subsequently assessed through role-play–based simulations.

The intervention content adopted a modular design, focusing on the development of 4 core psychological abilities for pregnant and postpartum women: stress regulation, problem-solving ability, behavioral activation, and social support ability. The intervention process followed a gradual approach and was implemented in 5 stages: the first stage focused on building a good therapeutic relationship, helping participants understand their responses to adversity and master basic relaxation techniques; the second stage systematically trained problem-solving skills, using methods such as brainstorming to find feasible solutions; the third stage used behavioral experiments to verify the effectiveness of the solutions and established a regular activity pattern; the fourth stage strengthened the social support network and improved the quality of interpersonal interactions; and the final stage involved an overall review and planning for future applications, ensuring the long-term maintenance of intervention effects. After each intervention, targeted homework assignments were given, and progress was monitored using standardized assessment tools. Detailed content is presented in Multimedia Appendix 1. Detailed problem management steps are presented in Multimedia Appendix 2.

To ensure the standardization and consistency of the intervention, the research team established a comprehensive quality control system, including audio recording analysis of 10% (n=17) of the intervention sessions, biweekly supervision meetings focusing on adherence and competence, and random quality checks. Participants who exhibited severe deterioration in mental health or were identified as having a risk of self-harm or suicide during the intervention period were immediately referred to a psychiatrist for specialized care and were withdrawn from the study.

### Control Group

The participants in the control group received a 5-week regimen of standard prenatal care, which was administered by obstetric nursing staff who underwent uniform training through a WeChat platform. The standard care program included weekly systematic health follow-ups, during which the nursing staff utilized standardized communication scripts to assess the physical and mental health status of the pregnant women. Additionally, professional prenatal health guidance was provided, covering basic health education services, such as prenatal examination precautions, key aspects of perinatal mental health maintenance, and newborn care knowledge.

### Outcomes

The primary outcome measure was depressive symptoms, assessed using the EPDS [26,27]. This scale consists of 10 items, with a 4-point Likert scale for scoring, where the frequency of symptoms is rated from “never” to “always,” corresponding to scores of 0-3. The Chinese version of the EPDS [28] demonstrates good internal reliability (Cronbach *α*=0.89). The clinical diagnostic criteria are as follows: a total score of less than 9 indicates a normal state, 9‐12 points suggest possible depression, and a total score of 13 or higher or a score greater than 0 on item 10 (self-harm thoughts) indicates a depressive state.

The following secondary outcome measures were also assessed: pregnancy-related anxiety: Pregnancy Anxiety Questionnaire (PAQ) [29]; perceived stress via the 4-item Perceived Stress Scale (PSS) [30,31]; and sleep quality via the Insomnia Severity Index (ISI) [32].

Additionally, baseline general information was collected, including age, gestational age, last menstrual period, height, current or prepregnancy weight, education level, marital status, occupation, residence, per capita family monthly income, living arrangements with elderly family members, knowledge of pregnancy-related information, and a history of prepregnancy depression. The data were collected at baseline (T1), immediately postintervention (T2), and 3 months postintervention (T3). The 3-month follow-up was selected, as it represents a standard medium-term evaluation period in behavioral intervention trials and aligns with prior WHO PM+ research, thereby facilitating cross-study comparison.

### Sample Size

The study employs the sample size estimation method for comparing the means of 2 samples, with depression scores as the calculation indicator, and uses the following formula to calculate the sample size:


(1) $$\begin{array}{c} n1=n2=2\times {\left[\left(\mu \alpha +\mu \beta \right)\;/d\right]}^{2} \end{array}$$

A 2-tailed test was adopted with *α*=.05 and *β*=.10. The anticipated effect size for this study was informed by the meta-analysis conducted by Ansaari et al [33] on online psychological interventions for perinatal depression, from which a Hedges *g* of 0.86 was derived for the primary outcome. Accounting for an estimated 20% loss to follow-up, the calculated sample size was approximately 37 participants per group, resulting in a total required sample of 74 participants. This attrition estimate is based on the typical range (15%‐25%) observed in comparable trials of PM+ [34].

### Statistical Analysis

Descriptive statistical analysis was used for baseline data: unordered categorical variables were expressed as percentages, and the chi-square test was used for between-group comparisons. When the expected count was less than 5, the Fisher exact test was applied. Ordinal categorical variables were described as median (IQR), and the Mann-Whitney *U* test was used for between-group comparisons. Normally distributed continuous variables were presented as mean (SD), with the independent samples *t*-test used for between-group comparisons; non-normally distributed continuous variables were described as median (IQR), and the Mann-Whitney *U* test was also used for between-group comparisons.

In this study, intention-to-treat analysis was adopted for primary and secondary outcomes. Based on the data after multiple imputation, linear mixed-effects models were used for analysis. The covariates included in the model were baseline variables with between-group differences (whether depression was present before pregnancy), and the random effect was set as the individual random intercept.

Standardized mean differences at time points T2 and T3 were calculated to represent between-group effect sizes, while standardized mean differences between T2, T3, and T1 within the same group served as within-group effect sizes. Both were quantified using Hedges *g* values and 95% CI. SPSS 26.0 (IBM Corp) was used for data analysis in this study, with the significance level set at *P*<.05.

## Results

### Overview

**Figure 1.**
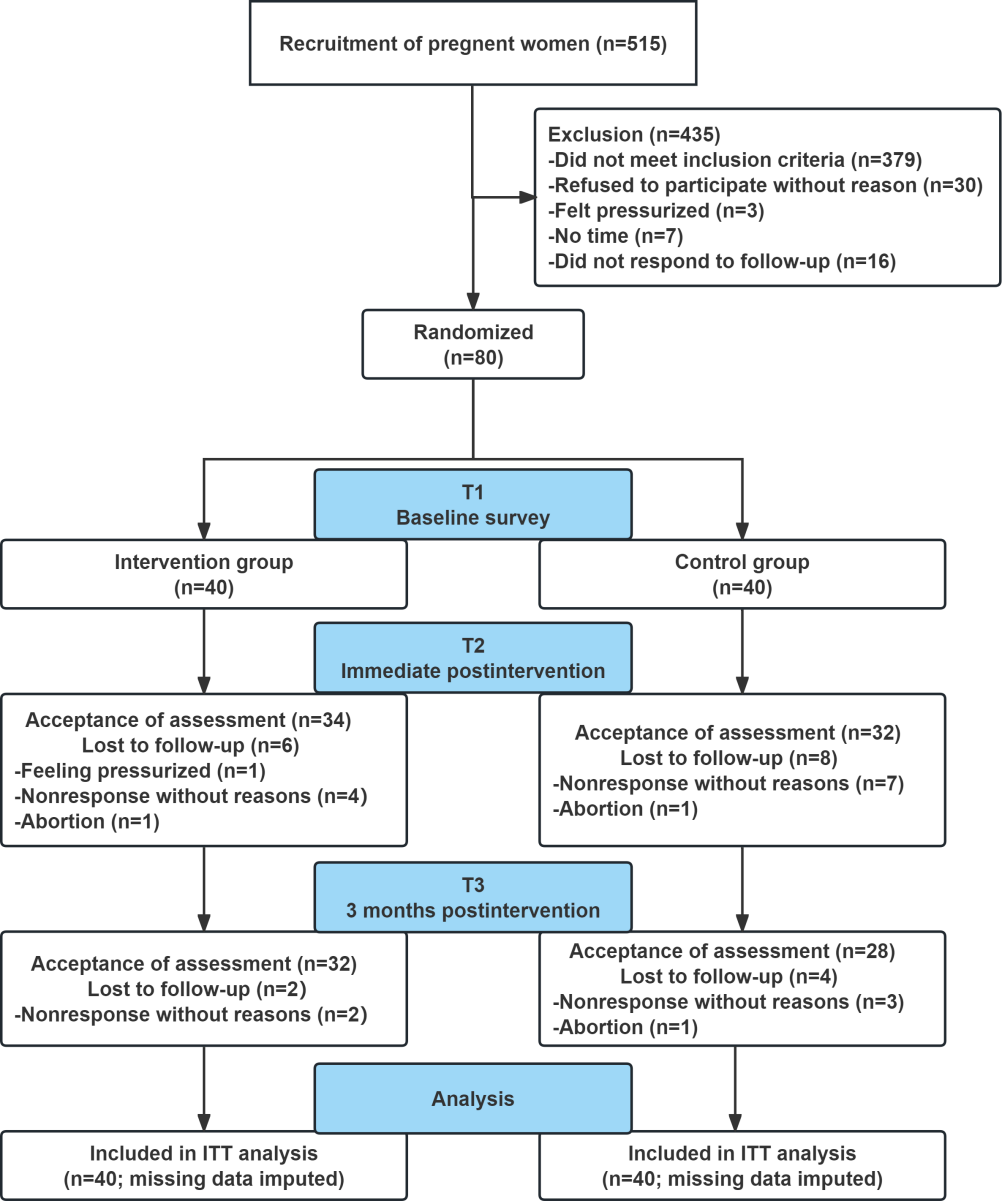
Flowchart of participants. ITT: intention-to-treat.

A total of 515 participants were screened for eligibility assessment between April 2024 and October 2024, and finally, 80 eligible participants were included in this study, who were randomly assigned to the intervention group (n=40) or the control group (n=40). In the intervention group, 34 (85.0%) participants and 32 (80%) participants completed the assessments at T2 and T3, respectively. In the control group, 32 (80%) participants and 28 (70.0%) participants completed the assessments at T2 and T3, respectively. In accordance with the intention-to-treat principle, all 80 randomized participants (40 per group) were included in the final analyses after missing data were handled using multiple imputation. The flowchart of participant recruitment and grouping is shown in Figure 1.

### Baseline Assessment

The baseline data of both the intervention group and the control group are presented in Table 1. A comparison of the 2 groups in terms of demographic characteristics revealed a statistically significant difference in the history of depression before pregnancy (*P*=.005), with the intervention group showing a higher proportion of individuals who had experienced depression prior to pregnancy. Regarding the scores of the EPDS, PAQ, PSS, and ISI, the differences between the 2 groups did not reach statistical significance.

**Table 1.** Baseline characteristics of participants from the intervention and control groups (n=80).

General information indicators	Intervention group (n=40)	Control group (n=40)	*P* value
Age (years), mean (SD)	30.63 (4.78)	30.43 (4.29)	.84
Gestational age, median (IQR)	13.5 (12.0-23.7)	14.5 (11.0-22.0)	.43
Number of pregnancies, n (%)			>.99
First pregnancy	24 (60)	24 (60)	
Nonfirst pregnancy	16 (40)	16 (40)	
Number of deliveries, n (%)			.45
Primiparous	31 (77.5)	28 (70.0)	
Multiparous	9 (22.5)	12 (30.0)	
Prepregnancy BMI (kg/m²), median (IQR)	22.0 (20.0-24.0)	21.0 (20.0-25.0)	.78
Baseline BMI (kg/m²), median (IQR)	23.0 (20.0-25.0)	22.5 (12.0-27.0)	.54
Employment status, n (%)			.45
Employed	28 (70)	31 (77.5)	
Other	12 (30)	9 (22.5)	
Place of residence, n (%)			.53
Rural	5 (12.5)	7 (17.5)	
Urban or city	35 (87.5)	33 (82.5)	
Whether living with elderly family members, n (%)			.50
Yes	20 (50)	23 (57.5)	
No	20 (50)	17 (42.5)	
History of depression before pregnancy, n (%)			.005
Yes	8 (20)	0 (0)	
No	32 (80)	40 (100)	
Education level, n (%)			.05
Elementary school or below	0 (0)	0 (0)	
Middle school	1 (2.5)	5 (12.5)	
High school or vocational school	8 (20)	10 (25.0)	
Bachelor’s degree	22 (55.0)	21 (52.5)	
Master’s degree or higher	9 (22.5)	4 (10)	
Per capita monthly family income (yuan [US $]/month), n (%)			.52
3000-5000 [432-720]	7 (17.5)	7 (17.5)	
5001-10,000 [720-1440]	17 (42.5)	19 (47.5)	
>10,000 [>1440]	14 (35)	11 (27.5)	
Knowledge level of pregnancy, n (%)			.75
Not at all knowledgeable	3 (7.5)	3 (7.5)	
Somewhat knowledgeable	35 (87.5)	36 (90)	
Very knowledgeable	2 (5.0)	1 (2.5)	
Depressive symptoms (EPDS^a^), mean (SD)	12.32 (1.62)	11.85 (2.14)	.07
Anxiety symptoms (PAQ^b^), mean (SD)	25.85 (2.92)	26.00 (4.25)	.89
Stress level (PSS^c^), mean (SD)	7.40 (1.94)	7.97 (1.34)	.47
Sleep quality (ISI^d^), mean (SD)	10.60 (5.65)	11.32 (4.89)	.66

a EPDS: Edinburgh Postnatal Depression Scale.

b PAQ: Pregnancy Anxiety Questionnaire.

c PSS: 4-item Perceived Stress Scale.

d ISI: Insomnia Severity Index.

### Outcome Measures

The linear mixed model uses the total score of the EPDS, the score of the PAQ, the score of the PSS, and the score of the ISI as dependent variables, with the control group and baseline time point (T1) as reference levels. It includes group (intervention group and control group), time (T1 or T2 or T3), and group×time interaction term as fixed effects, and controls for the covariate of a history of depression before pregnancy.

### Depressive Mood

Table 2 shows that the between-group effect (*F*_1,95.747_*=*13.564; *P<*.001), time effect (*F*_2,97.600_=116.563; *P<*.001), and group×time interaction effect (*F*_2,97.600_=24.203; *P*<.001) of depressive mood were all statistically significant.

**Table 2.** Results of fixed effects tests for each dependent variable.^a^

Effect type		*F* test (*df*)	*P* value
EPDS^b^			
	Between-group effect	13.564 (1, 95.747)	<.001
	Time effect	116.563 (2, 97.600)	<.001
	Interaction effect (time × between-group)	24.203 (2, 97.600)	<.001
PAQ^c^			
	Between-group effect	8.388 (1, 69.106)	.005
	Time effect	30.762 (2, 100.407)	<.001
	Interaction effect (time × between-group)	7.122 (2, 100.407)	.001
PSS^d^			
	Between-group effect	20.444 (1, 75.596)	<.001
	Time effect	29.707 (2, 90.567)	<.001
	Interaction effect (time × between-group)	7.255 (2, 90.567)	.001
ISI^e^			
	Between-group effect	13.458 (1, 73.646)	<.001
	Time effect	37.581 (2, 97.521)	<.001
	Interaction effect (time × between-group)	5.566 (2, 97.521)	.005

a The control group, T1 (baseline), and a history of depression before pregnancy were used as the reference categories.

b EPDS: Edinburgh Postnatal Depression Scale.

c PAQ: Pregnancy Anxiety Questionnaire.

d PSS: 4-item Perceived Stress Scale.

e ISI: Insomnia Severity Index.

The fixed-effects model (Table 3) analysis showed that the baseline effect in the intervention group was not statistically significant (*β*=.55; *P*=.24). The time effects were statistically significant at both T2 (*β*=−2.04; *P*<.001, 95% CI −2.92 to −1.16) and T3 (*β*=−2.34; *P*<.001, 95% CI −3.51 to −1.18) versus T1. We observed significant time×group interactions at T2 (*β*=−3.26; *P*<.001, 95% CI −4.51 to −2.01) and T3 (*β*=−3.75; *P*<.001, 95% CI −5.34 to −2.17) versus the control group T1. No history of depression before pregnancy showed an effect coefficient of 0.33 (*P*=.68; 95% CI −1.21 to 1.87), with no statistical significance. We reanalyzed the participants without a history of depression (32 participants in the intervention group and 40 participants in the control group). The results showed that the impact of the intervention on the primary outcomes (EPDS) of T2 (*β*=−3.40; *P*<.001) and T3 (*β*=−3.76; *P*<.001) remained very significant.

**Table 3.** Fixed effect estimation results of depression.^a^

Parameter	Coefficient (β)	SE	*t* test (*df*)	*P* value	95% CI	
					Lower bound	Upper bound
Intercept	12.00	0.91	13.15 (73.2)	<.001	10.21	13.79
Intervention group	0.55	0.47	1.17 (69.6)	.24	−0.37	1.46
History of depression before pregnancy						
No	0.33	0.79	0.42 (72.2)	.68	−1.21	1.87
Time						
T2	−2.04	0.45	−4.57 (87.8)	<.001	−2.92	−1.16
T3	−2.34	0.59	−3.99 (85.2)	<.001	−3.51	−1.18
Time×group interaction						
T2	−3.26	0.64	−5.12 (87.8)	<.001	−4.51	−2.01
T3	−3.75	0.80	−4.68 (85.2)	<.001	−5.34	−2.17

a The intervention group was referenced to the control group. Time points T2 and T3 were referenced to time point T1. The group without a history of depression before pregnancy was referenced to the group with a history of depression before pregnancy.

**Figure 2.**
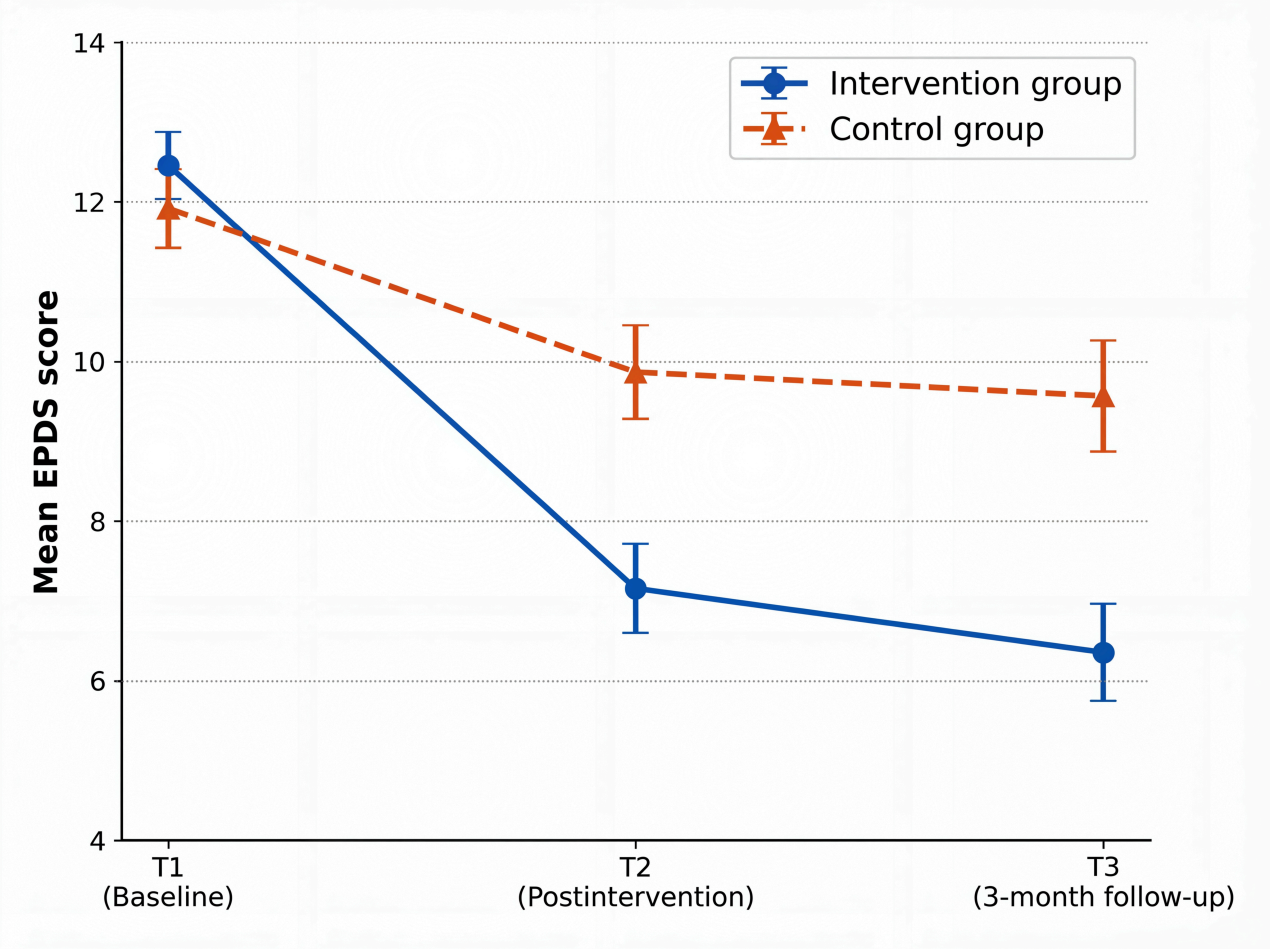
Line chart of the mean scores of Edinburgh Postnatal Depression Scale (EPDS) total scores in the 2 groups at 3 time points.

The trend of mean EPDS total scores over the 3 time points in the intervention group and the control group is shown in Figure 2. The score trajectories of the 2 groups diverged immediately after the intervention (T2). The scores in the intervention group decreased sharply at T2 and remained stable at T3, whereas the scores in the control group showed only a modest decline and remained consistently higher than those in the intervention group.

Further intra- and intergroup effect comparisons were conducted. The analysis of intragroup effect sizes (Table 4) showed that the intervention group exhibited significant reductions in depressive scores at T2 (Hedges *g*=1.69, 95% CI 1.18-2.20) and T3 (Hedges *g*=1.75, 95% CI 1.23-2.26), with large effect sizes. In contrast, the reduction in the control group was smaller (T2: Hedges *g*=0.58, 95% CI 0.13-1.02; T3: Hedges *g*=0.59, 95% CI 0.13-1.03). In intergroup comparisons, the intervention group scored significantly lower than the control group in terms of depression scores at T2 (Hedges *g*=0.74, 95% CI 0.23-1.24) and T3 (Hedges *g*=0.76, 95% CI 0.25-1.28; Table 4, Figure 2).

**Table 4.** Between-group and within-group effect sizes.^a^

	Intervention group (n=40)			Control group (n=40)			Between-group	
	Mean (SD)	Hedges *g* (95% CI)	*P* value	Mean (SD)	Hedges *g* (95% CI)	*P* value	Hedges *g* (95% CI)	*P* value
EPDS^b^								
T1	12.46 (2.67)	—^c^	—	11.92 (3.11)	—	—	—	—
T2	7.16 (3.51)	1.69 (1.18 to 2.20)	<.001	9.87 (3.71)	0.58 (0.13 to 1.02)	.003	0.74 (0.23 to 1.24)	.001
T3	6.36 (3.85)	1.75 (1.23 to 2.26)	<.001	9.57 (4.40)	0.59 (0.13 to 1.03)	<.001	0.76 (0.25 to 1.28)	<.001
PAQ^d^								
T1	24.72 (5.34)	—	—	24.89 (6.07)	—	—	—	—
T2	19.82 (5.06)	0.91 (0.44 to 1.37)	<.001	22.70 (6.13)	0.35 (0.06 to 0.62)	.008	0.51 (0.03 to 0.98)	.03
T3	20.54 (5.27)	0.77 (0.31 to 1.23)	<.001	23.56 (6.05)	0.21 (−0.03 to 0.45)	.08	0.52 (0.04 to 0.99)	.02
PSS^e^								
T1	7.65 (2.25)	—	—	8.33 (2.61)	—	—	—	—
T2	5.85 (2.51)	0.74 (0.27 to 1.22)	<.001	7.83 (2.96)	0.17 (−0.14 to 0.48)	.20	0.71 (0.23 to 1.18)	.002
T3	5.50 (2.70)	0.82 (0.34 to 1.31)	<.001	7.34 (3.01)	0.34 (−0.07 to 0.75)	.09	0.62 (0.15 to 1.11)	.005
ISI^f^								
T1	9.83 (4.36)	—	—	10.54 (4.91)	—	—	—	—
T2	5.06 (4.71)	1.01 (0.46 to 1.57)	<.001	7.26 (5.44)	0.61 (0.13 to 1.09)	.002	0.42 (−0.08 to 0.94)	.06
T3	4.88 (4.86)	1.03 (0.48 to 1.59)	<.001	8.52 (5.51)	0.37 (−0.03 to 0.79)	.079	0.68 (0.19 to 1.17)	.002

a Small, medium, and large effect sizes are defined as 0.2, 0.5, and 0.8, respectively. Lower scores indicate lower levels of depression. Statistical significance is determined when the 95% CI of Hedges *g* does not include "0." T1 is used as the reference.

b EPDS: Edinburgh Postnatal Depression Scale.

c Not applicable.

d PAQ: Pregnancy Anxiety Questionnaire.

e PSS: 4-item Perceived Stress Scale.

f ISI: Insomnia Severity Index.

### Secondary Outcome

Among the secondary outcome indicators, the between-group effect (*F*_1,69.106_=8.388; *P*=.005), time effect (*F*_2,100.407_=30.762; *P*<.001), and interaction effect (*F*_2,100.407_=7.122; *P*=.001) of anxiety symptoms; the between-group effect (*F*_1,75.596_=20.444; *P*<.001), time effect (*F*_2,90.567_=29.707; *P*<.001), and interaction effect (*F*_2,90.567_=7.255; *P*=.001) of perceived stress; and the between-group effect (*F*_1,73.646_=13.458; *P*<.001), time effect (*F*_2,97.521_=37.581; *P*<.001), and interaction effect (*F*_2,97.521_=5.566; *P*=.005) of sleep quality were all statistically significant (Table 2).

The results of fixed-effect estimates (Multimedia Appendix 3) revealed the dynamic change characteristics of secondary outcomes. For anxiety symptoms (Table S3 in Multimedia Appendix 3), the baseline levels were comparable between the 2 groups (*β*=−0.17*, t*_71.2_=−0.17; *P*=.87). The time effect was significant at T2 (*β*=−2.19*, t*_107.5_=−3.18; *P*<.001) and marginally significant at T3 (*β*=−1.33, *t*_101.0_=−1.95; *P*=.05). Additionally, the group×time interaction effects were significant at both T2 (*β*=−2.72*, t*_107.5_=−2.78; *P*=.006) and T3 (*β*=−2.86*, t*_101.0_=−2.99; *P*=.003), suggesting that the intervention group had more prominent improvements in anxiety symptoms.

Regarding perceived stress (Table S4 in Multimedia Appendix 3), there was no significant difference in baseline levels between the 2 groups (*β*=−.68*, t*_74.8_=−1.67*; P*=.096). The time effect was significant at T3 (*β*=−.98*, t*_88.4_=−2.73; *P*=.007) but not at T2 (*β*=−0.49, *t*_100.7_=−1.52; *P*=.14). The interaction effect showed that the reduction in the PSS in the intervention group during period T2 was significantly greater than that in the control group (*β*=−1.30, *t*_100.7_=−3.17; *P*=.002); the between-group difference persisted during period T3 (*β*=−1.16, *t*_88.4_=−2.19; *P*=.03), but the effect intensity was slightly weaker than that at the T2 time point.

The fixed-effect estimates for sleep quality (Table S5 in Multimedia Appendix 3) indicated that the baseline levels were comparable between the 2 groups (*β*=−.71, *t*_54.2_=−0.87, *P=.*38). The time effects were significant at both T2 (*β*=−3.27, *t*_98.3_=−4.01; *P*<.001) and T3 (*β*=−2.01, *t*_118.1_=−2.66; *P*=.008), showing a steady improvement in sleep quality over time. Moreover, the group×time interaction effect was significant only at T3 (*β*=−2.93, *t*_118.1_=−2.72; *P*=.007).

**Figure 3.**
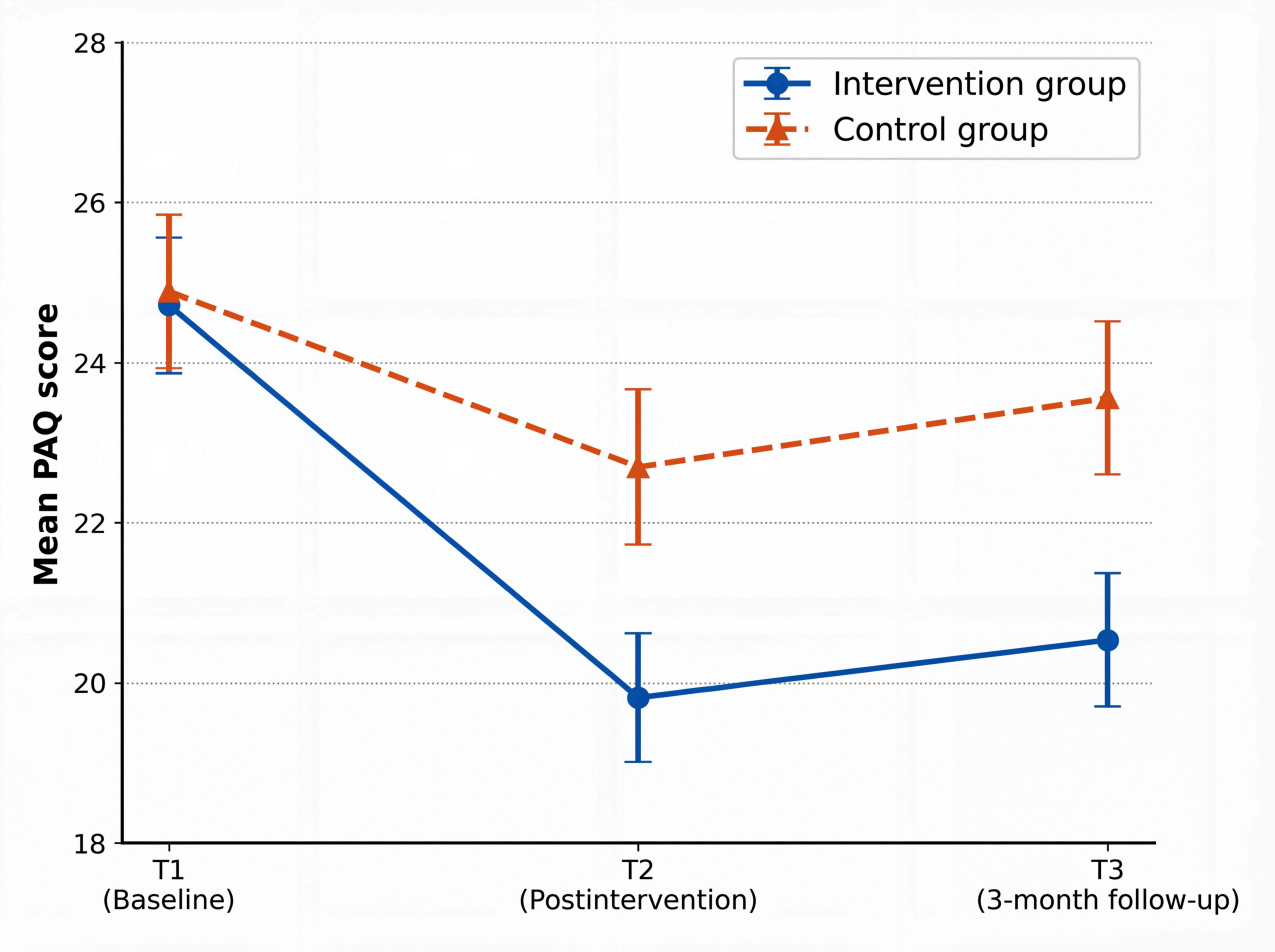
Line chart of the mean scores of Pregnancy Anxiety Questionnaire (PAQ) total scores in the 2 groups at 3 time points.

**Figure 4.**
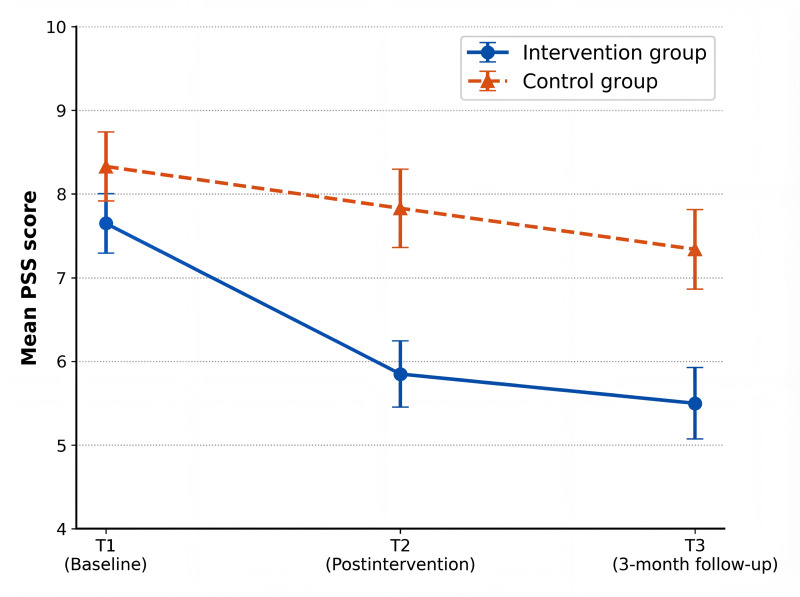
Line chart of the mean scores of the 4-item Perceived Stress Scale (PSS) total scores in the 2 groups at 3 time points.

**Figure 5.**
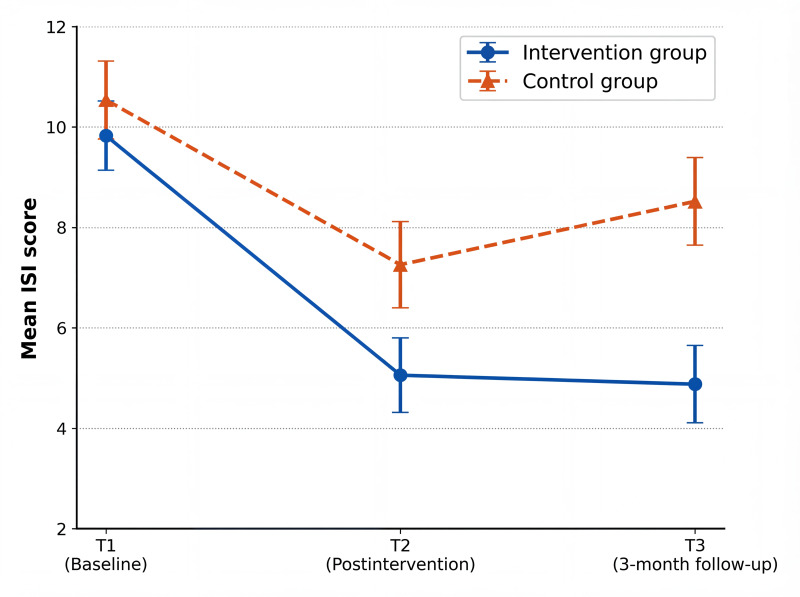
Line chart of the mean scores of Insomnia Severity Index (ISI) total scores in the 2 groups at 3 time points.

Line plots (Figures 3-5) depict the trajectories of anxiety, perceived stress, and sleep quality across the 3 assessment time points. In the intervention group, anxiety decreased markedly after the intervention (T2) and showed a slight rebound at follow-up (T3) while remaining well below baseline levels, whereas the control group exhibited only minor fluctuations over time. Perceived stress in the intervention group declined substantially at T2 and was maintained at follow-up, indicating sustained effects, while stress levels in the control group remained largely stable throughout the study. Differences in sleep quality between the groups were the smallest immediately postintervention (T2); however, a clear divergence emerged at follow-up (T3), with continued improvement observed in the intervention group and a rebound trend in the control group.

In terms of between-group effect sizes (Table 4), for anxiety symptoms, the scores of the intervention group at T2 (Hedges *g*=0.51, 95% CI 0.03-0.98) and T3 (Hedges *g*=0.52, 95% CI 0.04-0.99) were significantly lower than those of the control group, showing a medium effect. For perceived stress, the scores of the intervention group at T2 (Hedges *g*=0.71, 95% CI 0.23-1.18) and T3 (Hedges *g*=0.62, 95% CI 0.15-1.11) were significantly lower than those of the control group, presenting a medium effect. For sleep quality, the score of the intervention group at T3 (Hedges *g*=0.68, 95% CI 0.19-1.17) was significantly lower than that of the control group, with a medium effect, while the effect size at T2 was 0.42 (95% CI –0.08 to 0.94), which was not statistically significant.

### Sensitivity Analysis

To assess the potential impact of missing data on the results, this study conducted a linear mixed-effects model analysis on the original un-imputed data and compared the effect sizes (Hedges *g*), direction, magnitude, confidence intervals (95% CI), and significance levels (*P*-values) before and after multiple imputation. The results showed that the missing data did not alter the significance or direction of the core outcome measures, supporting the robustness of the study’s conclusions. The detailed results are presented in Table S6 in Multimedia Appendix 3.

### Safety and Adverse Events

No serious adverse events related to the intervention were reported throughout the trial. In the intervention group, 1 participant withdrew at T2 due to feeling stressed by the pace of the program, which was recorded as a nonserious intervention–related adverse event. Another participant withdrew because of a miscarriage; this event was determined to be unrelated to the intervention. No participants required referral to psychiatric services due to acute suicidal risk or severe deterioration in mental health.

## Discussion

### Principal Findings

To our knowledge, this study represents the first randomized controlled trial in a Chinese cultural context to examine the effectiveness of an IPM+ intervention for antenatal depression. The results demonstrate that IPM+ was associated with significant and sustained improvements in depressive symptoms, anxiety, and perceived stress up to 3 months after the intervention. Notably, improvements in sleep quality exhibited a delayed pattern, with clearer benefits observed at follow-up (T3).

### The Intervention Effect of IPM+ on Prenatal Depressive Mood

The results of this study demonstrate that the IPM+ intervention effectively alleviated AND, with effects sustained up to 3 months postintervention, consistent with the findings by Fink et al [35] showing prolonged benefits of telephone-delivered PM+ on maternal psychological distress. The significant efficacy observed in this study may stem from the synergistic intervention of the IPM+ modular design on the multidimensional causes of perinatal depression, including stress management techniques that regulate physiological stress responses, problem-solving strategies that enhance perceived control and self-efficacy, behavioral activation that counters low mood and social withdrawal, and social support facilitation that buffers stress and isolation. By simultaneously targeting the physiological, cognitive, behavioral, and social dimensions of depression, IPM+ may achieve sustained benefits within a relatively brief intervention period of 5 weeks. Notably, despite a higher proportion of participants with a history of prepregnancy depression in the intervention group, analyses indicated that prepregnancy depressive history did not significantly influence improvements in depressive symptoms, suggesting that IPM+ may be broadly applicable across perinatal women with varying levels of depression risk.

Compared with internet-based cognitive behavioral therapy (iCBT), IPM+ appears to be more time-efficient. A meta-analysis by Pan et al [36] indicated that interventions lasting fewer than 9 weeks did not produce significant improvements in postnatal depression (standardized mean difference=−0.36; *P*=.06), whereas the interventions of 9 weeks or longer demonstrated large and significant effects (standardized mean difference=−0.85; *P*<.01). In contrast, the IPM+ intervention achieved significant reductions in depressive symptoms within only 5 weeks, thereby reducing participant time burden and potentially enhancing the accessibility of psychological interventions for perinatal women. From a cost perspective, the IPM+ allows for delivery by nonspecialists, offering a scalable and economical alternative to therapist-intensive models, such as iCBT. This is particularly vital for implementation in resource-constrained settings.

Relative to existing internet-based mindfulness-based interventions, IPM+ also demonstrated comparatively larger effect sizes. For example, a randomized controlled trial by Kim et al. [37] reported moderate effects of a mobile-based mindfulness intervention on depressive severity (partial *η*²=0.06), whereas this study observed a large effect size for perinatal depression. This disparity likely stems from the divergent mechanisms underlying each intervention. While internet-based mindfulness-based interventions primarily foster the awareness and acceptance of emotional distress, IPM+ is anchored in structured problem-solving—a framework that directly targets the concrete, real-world challenges inherent to the perinatal period, such as role transitions and shifting relationship dynamics. By equipping women to actively resolve these tangible stressors, IPM+ appears mechanistically better aligned with the needs of this population, ultimately driving the larger effect sizes observed in our study.

Furthermore, although IPT is well established as an effective treatment, IPM+ offers notable advantages in scalability. A systematic review by Couch et al [38] showed that IPT relies almost exclusively on specialist providers and typically requires 8‐14 sessions, limiting its large-scale implementation in resource-constrained settings. By contrast, the structured 5-week IPM+ program can be delivered by nonspecialist providers, substantially lowering the threshold for dissemination of depression interventions.

### The Intervention Effect of IPM+ on Anxiety, Perceived Stress, and Sleep

This study further demonstrates that the IPM+ intervention exerted significant and sustained positive effects on anxiety and perceived stress among pregnant women, supporting its effectiveness as a transdiagnostic intervention recommended by the WHO. The intervention group showed sharp reductions in anxiety (PAQ) and stress scores immediately after the intervention (T2), with effects maintained at a moderate magnitude at the 3-month follow-up (T3). This pattern of rapid onset and durable improvement is highly consistent with the findings from face-to-face PM+ trials reported by Dawson et al [39] and Jordans et al [40], which showed superiority over usual care, providing strong evidence that the therapeutic effects of PM+ remain robust when extended to digital delivery formats. The marked improvements in anxiety and stress are likely attributable to the synergistic effects of the stress management and problem-solving modules, which may confer advantages over mindfulness-based interventions that primarily emphasize emotional awareness [41] or iCBT approaches that focus on cognitive restructuring [42] when addressing the unique stressors of the perinatal period. The stress management module offers relaxation techniques aimed at regulating heightened physiological arousal, consistent with established principles of relaxation training for anxiety management [11]. Importantly, the problem-solving module guides participants through structured steps to transform diffuse anxiety-provoking worries—such as excessive concerns about fetal health—into concrete, manageable problems with actionable plans. This process may directly target core cognitive maintenance factors of anxiety and stress, including intolerance of uncertainty and avoidance. The sustained reduction in stress observed at follow-up may further reflect the continued application of problem-solving skills in daily life, enhancing perceived control, alongside the facilitative role of the social support module in mobilizing available resources.

Sleep quality exhibited a distinct pattern of delayed improvement in our study, which contrasts with the findings from a CBT for insomnia study on perinatal insomnia by Quin et al [43], where interventions directly targeting sleep-related behaviors typically led to immediate postintervention improvements. The therapeutic pathway of PM+ may involve initial reductions in daytime emotional burden and cognitive rumination through the stress management and problem-solving modules, leading to decreased nocturnal psychophysiological arousal. Building on this foundation, the behavioral activation module may contribute to more regular daily routines and increased daytime activity, thereby supporting circadian rhythm stabilization, while the social support module may alleviate feelings of loneliness and burden during pregnancy by strengthening social connectedness. Together, these changes form a gradual cycle of “reduced daytime emotional distress → enhanced nighttime calmness → progressive establishment of healthier sleep patterns.” This cycle likely requires several weeks to consolidate into measurable sleep improvements, providing a plausible explanation for why sleep-related effects were not fully evident immediately postintervention (T2) but became significant at follow-up (T3). This finding aligns with emerging evidence highlighting emotion regulation as a key mechanism underlying improvements in comorbid insomnia [44].

### Limitations and Suggestions

This study has several limitations that should be acknowledged. First, the sample size is relatively small (n=80) and of limited representativeness. Participants were recruited from a single tertiary hospital in Wuhan, where the levels of education and access to health care are generally high. Therefore, the generalizability of the study findings to the maternal population in rural areas or primary health care institutions may be limited. Future studies should adopt multicenter designs with larger, more diverse samples that include women from different geographic regions, health care tiers, and socioeconomic backgrounds to better evaluate the generalizability of IPM+. Second, we did not assess participants’ blinding success after the intervention or examine whether group guessing influenced self-reported outcomes. Future studies could systematically evaluate participants’ group guesses after the intervention and include this as a covariate in analyses to control for potential expectation effects or reporting bias. Third, symptom outcomes were primarily measured using self-report measures without confirmation via structured clinical interviews based on diagnostic criteria. Combining self-report scales with standardized diagnostic interviews in future work would help mitigate subjective bias and strengthen clinical validity. Finally, the 3-month follow-up period limits conclusions regarding the longer-term impact of IPM+ on postnatal mental health and mother-infant outcomes. Future studies with extended follow-up windows are needed and should include more ecologically meaningful endpoints, such as maternal-infant attachment quality and infant emotional-behavioral development.

This study presents a scalable framework for integrating perinatal mental health support into China’s existing health care infrastructure. In the future, the core modules of IPM+ could be integrated into hospital-based official online service platforms as a universal and preventive psychological support tool for all pregnant women, facilitating early identification and timely intervention. In addition, the task-shifting potential of PM+ could be further leveraged through standardized training and supervision of maternal and child health providers in community health centers, enabling the delivery of preliminary yet fidelity-consistent psychological support at the primary care level. Such an approach may help alleviate the imbalance between the high demand for perinatal mental health services and the limited availability of specialist providers.

### Conclusion

The findings from this randomized controlled trial indicate that the IPM+ intervention significantly alleviated depressive and anxiety symptoms, reduced perceived stress, and improved sleep quality among pregnant women, with benefits sustained at the 3-month follow-up. By employing structured modules that simultaneously address perinatal-specific stressors and internal emotional distress, IPM+ establishes a targeted and practical support framework. These results support IPM+ as an effective, sustainable, and scalable approach to promoting perinatal mental health. Future implementation should focus on integrating IPM+ into routine antenatal care pathways to enhance accessibility and impact.

## Supplementary material

10.2196/81998 Multimedia Appendix 1 The problem management plus (PM+) group intervention program.

10.2196/81998 Multimedia Appendix 2 Problem management steps.

10.2196/81998 Multimedia Appendix 3 Fixed effect estimation results of anxiety, perceived stress, and sleep quality; Comparison of analysis results before and after multiple imputation.

10.2196/81998 Checklist 1 CONSORT checklist.

## Abbreviations

AND: antenatal depression
CBT: cognitive behavioral therapy
CONSORT: Consolidated Standards of Reporting Trials
EPDS: Edinburgh Postnatal Depression Scale
iCBT: internet-based cognitive behavioral therapy
IPM+: internet-based problem management plus
IPT: interpersonal psychotherapy
ISI: Insomnia Severity Index
PAQ: Pregnancy Anxiety Questionnaire
PM+: problem management plus
PSS: 4-item Perceived Stress Scale
WHO: World Health Organization
