# Supplementary material for: Internet-Based Problem Management Plus Intervention for Antenatal Depression: Randomized Controlled Trial
Source: J Med Internet Res. 2026 Mar 27;28:e81998. doi: 10.2196/81998 (PMC13026447; doi:10.2196/81998)
Supplement: Multimedia Appendix 1 [file jmir-v28-e81998-s001.docx]

## Table S1. The PM+ Group Intervention Program

| Time | Theme | Goal | Intervention content |
| --- | --- | --- | --- |
| Week 1 | ①Understand adversity, understand PM+  ② Stress Management | ①Help service recipients understand and participate in PM+ and normalize their responses when facing difficulties and stress  ②Help pregnant women relax and manage psychophysiological issues caused by stress | ① Brief introduction to PM+ and confidentiality agreement (5 minutes): Introduce the content, duration, and pros/cons of participating in the "PM+" intervention to pregnant women, and establish trust.  ② Review based on assessment and pre-session evaluation (10 minutes): Understand the difficulties they have faced in the past week to facilitate further discussion.  ③ What is PM+ (20 minutes): Help pregnant women understand and engage with PM+.  ④ Understanding adversity (30 minutes): Help pregnant women understand the adversity they face and normalize their reactions to difficulties and stresses (fetal health anxiety and childbirth fear, among others).  ⑤ Learning and mastering stress management techniques (20 minutes): Slow breathing in a sitting or side-lying position (the primary practice strategy), yoga, etc.  ⑥ Setting practice tasks and concluding the session (5 minutes): Distribute stress management worksheets and weekly schedules, encouraging pregnant women to practice slow breathing every day before the second session until they become accustomed to using slow breathing to relieve stress in daily life |

**Table S1  (continued). The PM+ Group Intervention Program**

| Time | Theme | Goal | Intervention content |
| --- | --- | --- | --- |
| Week 2 | Problem Management | Improve pregnant women’s ability to manage and solve problems | ① Overall review and pre - course assessment (5 minutes): Review the overall situation of the past week and discuss the practice of stress - reduction techniques such as slow breathing from last week.  ② Problem management (70 minutes): This module is mainly used to solve some practical problems encountered by pregnant women. During the intervention, the intervenor and the pregnant women work together to come up with feasible solutions to the problems they are most concerned about. For details, see Annex 2.  ③ Practice "stress management": Consolidate the practice of slow breathing strategies.  ④ Set practice tasks and end the course (5 minutes): Encourage pregnant women to insist on practicing slow breathing every day before the third meeting, write down the problem - solving methods obtained from the brainstorming on the weekly schedule, and strictly implement these solutions. |
|  |  |  |  |

**Table S1  (continued). The PM+ Group Intervention Program**

| Time | Theme | Goal | Intervention content |
| --- | --- | --- | --- |
| Week 3 | Behavioral Activation | Break the vicious cycle of inactivity and low mood, and encourage pregnant women to re-engage in pleasant, task-oriented activities to improve their mood | ① Overall review and pre-course assessment (5 minutes): Review the overall situation of the past week and explore whether the problems from last week have been resolved.  ② Review of "Problem Management" (35 minutes): Discuss with pregnant women the progress of the problem management action plan, the problems encountered, and the next arrangements.  ③ Take action and persevere (35 minutes): Break the vicious cycle of inactivity → low mood → reluctance to engage in activities. Encourage pregnant women to re-participate in pleasant, task-oriented activities, such as a reasonable diet, regular exercise, reading, meditation, etc.; break down tasks into small steps; arrange specific times to complete the activities; use the mobile phone reminder function to prompt pregnant women to complete them, so as to improve their mood.  ④ Practice "Stress Management" (10 minutes): Same as above.  ⑤ Set practice tasks and end the course (5 minutes): Distribute the "Behavioral Activation" information sheet, and encourage pregnant women to practice the problem management plan and behavioral activation activities. |

**Table S1  (continued). The PM+ Group Intervention Program**

| Time | Theme | Goal | Intervention content |
| --- | --- | --- | --- |
| Week 4 | Strengthening Social Support | Enhance social support for pregnant women and promote their overall emotional health | ① Overall review and pre-course assessment (5 minutes): Review the overall situation of the past week, explore whether the problems from last week have been resolved, and whether new problems have emerged.  ② Problem management (20 minutes): Conduct brainstorming on newly emerged problems.  ③ Review of "Behavioral Activation" (20 minutes): Review the progress of activities and difficulties encountered, determine new activities or tasks that pregnant women can complete within a week, or increase the frequency of activities they have already started participating in. Try to help pregnant women select a series of different types of tasks and activities (such as doing housework, socializing, exercising, etc.).  ④ Strengthening social support: Introduce to pregnant women the forms and benefits of social support; help them identify a person, community organization, or more formal support agency they are willing to seek help from, and assist them in determining when, where, and specifically what kind of help to seek; encourage pregnant women to proactively strengthen interactions with family members, friends, and colleagues in daily life through conversations, WeChat contacts, etc., to understand and help each other; join pregnant mothers' communication groups, and encourage them to share the troubles they face during pregnancy and pregnancy-related information with each other.  ⑤ Practice "Stress Management": Same as above.  ⑥ Set practice tasks and end the course: Before the fifth meeting, practice slow breathing every day, encourage pregnant women to find a trusted person to talk to about their troubles, and continue to complete the plan according to the schedule. |

**Table S1  (continued). The PM+ Group Intervention Program**

| Time | Theme | Goal | Intervention content |
| --- | --- | --- | --- |
| Week 5 | Maintain Health and Look to the Future | Help pregnant women prevent the recurrence of emotional problems and strengthen their confidence in continuing to use the "PM+" intervention strategy | ① Overall review (20 minutes): Discuss with pregnant women their experiences after completing all action plans.  ② Maintain health (30 minutes): Praise the efforts made by pregnant women in participating in this research, and encourage them to continue practicing these strategies to maintain a good state.  ③ Imagine how to help others (20 minutes): Take pregnant women's relatives and friends as examples, and think about what strategies to use to help them if they are facing problems such as unemployment, illness or broken relationships.  ④ Look to the future (15 minutes): Discuss the goals of maintaining emotional health in the future and plan how to achieve them.  ⑤ End the course (5 minutes): Encourage pregnant women to practice slow breathing every day and actively use these strategies when they encounter difficulties in the future. |
