# Supplementary material for: Internet-Based Problem Management Plus Intervention for Antenatal Depression: Randomized Controlled Trial
Source: J Med Internet Res. 2026 Mar 27;28:e81998. doi: 10.2196/81998 (PMC13026447; doi:10.2196/81998)
Supplement: Multimedia Appendix 2 [file jmir-v28-e81998-s002.doc]

## **Table S2.** Problem Management Steps

| Step | Description |
| --- | --- |
| 1. List the problems | List "solvable" and "unsolvable" problems, that is, problems that can be changed and problems that cannot be changed. |
| 2. Select one problem | First, choose a relatively easy (solvable) problem. |
| 3. Define the problem | Select the elements in the problem that are of practical significance and can be controlled or influenced to a certain extent; The explanation of the problem should be as specific and concise as possible; Try to avoid including multiple problems; If a problem consists of multiple parts, it needs to be split and each part handled separately. |
| 4. Brainstorm | First, encourage pregnant women to come up with as many possible solutions to the problem as possible, and there is no need to consider whether the methods are good or bad at this stage; Let pregnant women think about what they can do and who can help them deal with part of the problem; Consider existing personal strengths, resources, or support; Try to encourage pregnant women to come up with solutions on their own instead of directly providing them (if you want to give advice, you need to first ask what they would say to a friend). |
| 5. Select and determine helpful solutions | From the listed solutions, select the ones that are most useful and can best change the problem; Useful methods need to be feasible; More than one solution can be chosen. |
| 6. Action plan | Formulate a detailed plan, including the way and time of implementation; It is recommended to use reminder tools to remind yourself to implement the plan, such as using notes or schedules, combining the time of meals or other activities when arranging activities, etc. |
| 7. Review | Discuss what they have done and its impact on the initial problem; Discuss the difficulties encountered in the implementation of the plan; Based on the progress of the plan completed last week, discuss and plan what can be done next week to continue to influence and manage the problem. |
