# Supplementary material for: Internet-Based Problem Management Plus Intervention for Antenatal Depression: Randomized Controlled Trial
Source: J Med Internet Res. 2026 Mar 27;28:e81998. doi: 10.2196/81998 (PMC13026447; doi:10.2196/81998)
Supplement: Multimedia Appendix 3 [file jmir-v28-e81998-s003.docx]

Table S3 Fixed effect estimation results of anxiety

| Parameter | Coefficient (β) | Standard error | *t* | *p* | 95% CI | |
| --- | --- | --- | --- | --- | --- | --- |
|  |  |  |  |  | Lower bound | Upper bound |
| Intercept | 24.94 | 1.68 | 14.829 | ＜0.001 | 21.64 | 28.24 |
| Intervention group | -0.17 | 0.98 | -0.17 | 0.865 | -2.10 | 1.76 |
| Time |  |  |  |  |  |  |
| T2 | -2.19 | 0.69 | -3.181 | 0.002 | -3.54 | -0.83 |
| T3 | -1.33 | 0.68 | -1.948 | 0.052 | -2.66 | 0.01 |
| History of depression before pregnancy |  |  |  |  |  |  |
| No | 1.62 | 1.40 | 1.157 | 0.247 | -1.12 | 4.36 |
| Time × Group interaction |  |  |  |  |  |  |
| T2 | -2.72 | 0.98 | -2.783 | 0.006 | -4.65 | -0.79 |
| T3 | -2.86 | 0.95 | -2.994 | 0.003 | -4.73 | -0.98 |

Table S4 Fixed effect estimation results of perceived stress

| Parameter | Coefficient (β) | Standard error | *t* | *p* | 95% CI | |
| --- | --- | --- | --- | --- | --- | --- |
|  |  |  |  |  | Lower bound | Upper bound |
| Intercept | 8.67 | 0.76 | 11.393 | ＜0.001 | 7.18 | 10.17 |
| Intervention group | -0.68 | 0.41 | -1.667 | 0.096 | -1.48 | 0.12 |
| Time |  |  |  |  |  |  |
| T2 | -0.49 | 0.33 | -1.516 | 0.135 | -1.15 | 0.16 |
| T3 | -0.98 | 0.36 | -2.733 | 0.007 | -1.69 | -0.28 |
| History of depression before pregnancy |  |  |  |  |  |  |
| No | -0.63 | 0.63 | -0.993 | 0.321 | -1.87 | 0.61 |
| Time × Group interaction |  |  |  |  |  |  |
| T2 | -1.30 | 0.41 | -3.172 | 0.002 | -2.10 | -0.49 |
| T3 | -1.16 | 0.53 | -2.188 | 0.031 | -2.21 | -0.11 |

Table S5 Fixed effect estimation results of sleep quality

| Parameter | Coefficient (β) | Standard error | *t* | *p* | 95% CI | |
| --- | --- | --- | --- | --- | --- | --- |
|  |  |  |  |  | Lower bound | Upper bound |
| Intercept | 10.62 | 1.38 | 7.713 | ＜0.001 | 7.92 | 13.32 |
| Intervention group | -0.71 | 0.81 | -0.873 | 0.383 | -2.30 | 0.88 |
| Time |  |  |  |  |  |  |
| T2 | -3.27 | 0.74 | -4.406 | ＜0.001 | -4.73 | -1.81 |
| T3 | -2.01 | 0.76 | -2.664 | 0.008 | -3.50 | -0.53 |
| History of depression before pregnancy |  |  |  |  |  |  |
| No | 1.28 | 1.15 | 1.12 | 0.263 | -0.96 | 3.53 |
| Time × Group interaction |  |  |  |  |  |  |
| T2 | -1.49 | 1.03 | -1.451 | 0.147 | -3.50 | 0.52 |
| T3 | -2.93 | 1.08 | -2.72 | 0.007 | -5.05 | -0.81 |

Table S6 Comparison of Analysis Results Before and After Multiple Imputation

| Outcome variable | Time | Type of analysis | Hedges'g (g) | Δg | 95% CI | p-value |
| --- | --- | --- | --- | --- | --- | --- |
| **EPDS** | **T2** | Before imputation | 0.78 | 0.04 | (0.27~1.28) | <0.001 |
|  |  | After imputation | 0.74 |  | (0.23~1.24) | <0.001 |
|  | **T3** | Before imputation | 0.91 | 0.15 | (0.39~1.41) | <0.001 |
|  |  | After imputation | 0.76 |  | (0.25~1.28) | <0.001 |
| **PAQ** | **T2** | Before imputation | 0.57 | 0.06 | (0.09~1.04) | <0.001 |
|  |  | After imputation | 0.51 |  | (0.03~0.98) | 0.006 |
|  | **T3** | Before imputation | 0.39 | 0.12 | (-0.01~0.81) | <0.001 |
|  |  | After imputation | 0.52 |  | (0.04~0.99) | 0.003 |
| **PSS** | **T2** | Before imputation | 0.85 | 0.14 | (0.28~1.41) | <0.001 |
|  |  | After imputation | 0.71 |  | (0.23~1.18) | 0.002 |
|  | **T3** | Before imputation | 0.74 | 0.12 | (0.18~1.30) | 0.005 |
|  |  | After imputation | 0.62 |  | (0.15~1.11) | 0.031 |
| **ISI** | **T2** | Before imputation | 0.44 | 0.02 | (-0.07~0.95) | 0.094 |
|  |  | After imputation | 0.42 |  | (-0.08~0.94) | 0.147 |
|  | **T3** | Before imputation | 0.80 | 0.12 | (0.23~1.36) | <0.001 |
|  |  | After imputation | 0.68 |  | (0.19~1.17) | 0.007 |
