# Supplementary material for: Internet-Based Problem Management Plus Intervention for Antenatal Depression: Randomized Controlled Trial
Source: J Med Internet Res. 2026 Mar 27;28:e81998. doi: 10.2196/81998 (PMC13026447; doi:10.2196/81998)
Supplement: Checklist 1 [file jmir-v28-e81998-s004.docx]

**CONSORT 2010 checklist of information to include when reporting a randomised trial***

| **Section/Topic** | **Item**  **No Checklist item** | **Reported**  **on page No** |
| --- | --- | --- |

**Title and abstract**

**Introduction**

Background and objectives

1a

1b

2a

2b

Identification as a randomised trial in the title

P1

| P1-2 |
| --- |
| P2-4 |
| P4 |
| P5 |

Structured summary of trial design, methods, results, and conclusions (for specific guidance see CONSORT for abstracts)

Scientific background and explanation of rationale Specific objectives or hypotheses


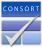
**Methods**

Description of trial design (such as parallel, factorial) including allocation ratio

Trial design

Participants

Interventions

Outcomes

3a

3b

4a

4b

5

6a

6b

7a

7b

8a

8b

9

10

11a

Important changes to methods after trial commencement (such as eligibility criteria), with reasons Eligibility criteria for participants

NA (No changes)

P5-6

Settings and locations where the data were collected

| P5 |
| --- |
| P7-9 |
| P9-10 |

The interventions for each group with sufficient details to allow replication, including how and when they were actually administered

Completely defined pre-specified primary and secondary outcome measures, including how and when they were assessed

Any changes to trial outcomes after the trial commenced, with reasons How sample size was determined

NA (No changes)

P10-11

Sample size

When applicable, explanation of any interim analyses and stopping guidelines

| NA |
| --- |
| P6 |
| P6 |
| P6 |
| P6 |

Randomisation:

Method used to generate the random allocation sequence

Sequence

Type of randomisation; details of any restriction (such as blocking and block size)

generation

Mechanism used to implement the random allocation sequence (such as sequentially numbered containers), describing any steps taken to conceal the sequence until interventions were assigned

Allocation

concealment

mechanism

Implementation

Who generated the random allocation sequence, who enrolled participants, and who assigned participants to interventions

If done, who was blinded after assignment to interventions (for example, participants, care providers, those

Blinding

Statistical methods

**Results**

Participant flow (a diagram is strongly recommended)

Recruitment

Baseline data

Numbers analysed

Outcomes and estimation

Ancillary analyses

Harms

**Discussion**

Limitations

Generalisability Interpretation

**Other information**

Registration Protocol

Funding

11b

12a

12b

13a

13b

14a

14b

15

16

17a

17b

18

19

20

21

22

23

24


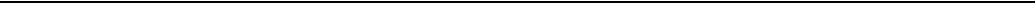
25

assessing outcomes) and how

If relevant, description of the similarity of interventions

Statistical methods used to compare groups for primary and secondary outcomes Methods for additional analyses, such as subgroup analyses and adjusted analyses

For each group, the numbers of participants who were randomly assigned, received intended treatment, and were analysed for the primary outcome

For each group, losses and exclusions after randomisation, together with reasons Dates defining the periods of recruitment and follow-up

Why the trial ended or was stopped

A table showing baseline demographic and clinical characteristics for each group

For each group, number of participants (denominator) included in each analysis and whether the analysis was by original assigned groups

For each primary and secondary outcome, results for each group, and the estimated effect size and its precision (such as 95% confidence interval)

For binary outcomes, presentation of both absolute and relative effect sizes is recommended

Results of any other analyses performed, including subgroup analyses and adjusted analyses, distinguishing pre-specified from exploratory

All important harms or unintended effects in each group (for specific guidance see CONSORT for harms)

Trial limitations, addressing sources of potential bias, imprecision, and, if relevant, multiplicity of analyses Generalisability (external validity, applicability) of the trial findings

Interpretation consistent with results, balancing benefits and harms, and considering other relevant evidence

Registration number and name of trial registry

Where the full trial protocol can be accessed, if available

Sources of funding and other support (such as supply of drugs), role of funders

P6

P6


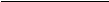
P11-12

P11-12

P12


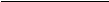

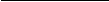
P12


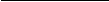
P12

NA

P13-14,Table 1


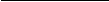
P12

Tables 2-4
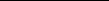


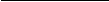
NA(No binary)


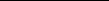
P16, 21-22


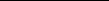
NA (No harm)

P27-28

| P27-28 |
| --- |
| P23-27 |
| P2 |
| Appendix1 |

P29


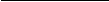

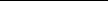

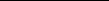

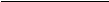

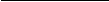
*We strongly recommend reading this statement in conjunction with the CONSORT 2010 Explanation and Elaboration for important clarifications on all the items. If relevant, we also

recommend reading CONSORT extensions for cluster randomised trials, non-inferiority and equivalence trials, non-pharmacological treatments, herbal interventions, and pragmatic trials. Additional extensions are forthcoming: for those and for up to date references relevant to this checklist, see www.consort-statement.org.
